# MG53 inhibits angiogenesis through regulating focal adhesion kinase signalling

**DOI:** 10.1111/jcmm.16777

**Published:** 2021-07-09

**Authors:** Jinling Dong, Haiyan Zhou, Yongjie Li, Rong Li, Ni Chen, Youkun Zheng, Xin Deng, Mao Luo, Jianbo Wu, Liqun Wang

**Affiliations:** ^1^ Drug Discovery Research Center Southwest Medical University Luzhou China; ^2^ Department of Pharmacology Laboratory for Cardiovascular Pharmacology The School of Pharmacy Southwest Medical University Luzhou China

**Keywords:** angiogenesis, focal adhesion kinase, Mitsugumin 53, Src

## Abstract

Mitsugumin 53 (MG53), which is expressed predominantly in striated muscle, has been demonstrated to be a myokine/cardiokine secreted from striated muscle under specific conditions. The important roles of MG53 in non‐striated muscle tissues have also been examined in multiple disease models. However, no previous study has implicated MG53 in the control of endothelial cell function. In order to explore the effects of MG53 on endothelial cells, human umbilical vein endothelial cells (HUVECs) were stimulated with recombinant human MG53 (rhMG53). Then, rhMG53 uptake, focal adhesion kinase (FAK)/Src/Akt/ERK1/2 signalling pathway activation, cell migration and tube formation were determined in vitro. The efficacy of rhMG53 in regulating angiogenesis was also detected in postnatal mouse retinas. The results demonstrated that rhMG53 directly entered into endothelial cells in a cholesterol‐dependent manner. The uptake of rhMG53 directly bound to FAK in endothelial cells, which resulted in a significant decrease in FAK phosphorylation at Y397. Accompanied by the dephosphorylation of FAK, rhMG53 uncoupled FAK‐Src interaction and reduced the phosphorylation of Src at Y416. Consequently, the activation of FAK/Src downstream signalling pathways, such as Akt and ERK1/2, was also significantly inhibited by rhMG53. Furthermore, rhMG53 remarkably decreased HUVEC migration and tube formation in vitro and postnatal mouse retinal angiogenesis in vivo. Taken together, these data indicate that rhMG53 inhibits angiogenesis through regulating FAK/Src/Akt/ERK1/2 signalling pathways. This may provide a novel molecular mechanism for the impaired angiogenesis in ischaemic diseases.

## 1 INTRODUCTION

Mitsugumin 53 (MG53), also known as TRIM72, belongs to tripartite motif (TRIM) protein family, which is composed of a prototypical TRIM domain (including a RING domain, a B‐box domain and a coiled‐coil domain) at its N terminus and a SPRY domain at its C terminus.^1^, ^2^ MG53 is expressed predominantly in striated muscle (cardiac and skeletal muscle)^3^ and has been reported to be involved in the regulation of a variety of physiological and pathological functions in striated muscle. One of the first discovered physiological functions of MG53 is involved in the repair of membrane damage.^3^ It is now well established that both endogenous and exogenous MG53 play important roles in the repair process of skeletal muscle, cardiac muscle and even other non‐striated muscle cell plasma membrane.^2^, ^4^, ^5^ Subsequently, it has been demonstrated that MG53 prevents myocardium from ischaemia/reperfusion (IR) injury through participating in ischaemic preconditioning and ischaemic postconditioning.^6^, ^7^

Meanwhile, the effects of MG53 on system metabolism regulation have been gradually elucidated, but are still controversial. The RING domain of MG53 is identified as a muscle‐specific E3 ubiquitin ligase that catalyses ubiquitin conjugation and mediates protein degradation.^8^, ^9^, ^10^, ^11^ It has been previously shown that endogenous MG53 negatively regulates insulin signalling via ubiquitination and degradation of insulin receptor‐β (IR‐β) and insulin receptor substrate‐1 (IRS‐1) in skeletal muscle.^9^, ^10^ Recombinant human MG53 (rhMG53) has also been demonstrated to allosterically inhibit insulin signalling through binding to the extracellular domain of IR‐β.^12^ These published reports indicate a causative role of MG53 in promoting the pathogenesis of diabetes. On the contrary, Ma and colleagues emphasize that neither sustained elevation of MG53 in circulation nor whole‐body ablation of MG53 has significant influences on glucose handling and insulin sensitivity in *db/db* mice,^13^, ^14^ which argues against the proposed function of MG53 as a causative factor for diabetes development.

Furthermore, growing evidence has also indicated the widely protective effects of rhMG53 on non‐striated muscle tissues in multiple disease models. Systemic administration of rhMG53 could reduce symptoms of lung injury via protecting impaired lung epithelial cells,^15^ ameliorate I/R‐, nephrotoxin‐ and cisplatin‐induced acute kidney injury via repairing injured renal proximal tubular epithelium cells,^16^ alleviate I/R‐induced brain injury via suppression of apoptotic neuronal cell death,^17^ decrease I/R‐induced liver injury via inhibiting oxidative stress and hepatocyte apoptosis,^18^ and promote the efficacy of human umbilical cord‐derived mesenchymal stem cells in the recovery of traumatic brain injury via promoting hUC‐MSC proliferation and migration.^19^ Moreover, the toxicology studies have certified that repetitive intravenous administration of rhMG53 does not result in a measurable impact on metabolic function or vital organ toxicity.^14^ These data highlight rhMG53 as a potentially safe biological reagent available for therapeutic application with no to limited effects on diabetes.

More importantly, previous studies have demonstrated that MG53 is myokine/cardiokine secreted from striated muscle in response to high glucose and high insulin, which may lead to a remarkable increase in serum MG53 content in rodents and humans with obesity and diabetes.^9^, ^12^, ^20^ As well as known, angiogenesis disorders, including both excessive and defective angiogenesis, generally exist in patients with diabetes, which contribute to the development of cardiovascular complications.^21^, ^22^ However, no previous studies have investigated whether the elevation of serum MG53 is involved in the impaired angiogenesis. Therefore, the aim of this study was to determine the effects of rhMG53 on angiogenesis and to understand the molecular mechanism involved.

Angiogenesis is a physiological process including the development of new blood vessels from pre‐existing vessels and the subsequent formation of a vascular network. The functional responses of endothelial cell, such as proliferation, migration and tube formation, are of great importance during angiogenesis. Focal adhesion kinase (FAK) is a cytoplasmic tyrosine kinase which is involved in integrin‐ and other cell surface receptor–mediated signal transductions. Previous studies have shown that the activation of FAK and its downstream signalling pathways, such as Src, Akt and ERK1/2, plays essential roles in cell adhesion, migration, tube formation and angiogenesis.^23^, ^24^, ^25^, ^26^ Nguyen et al have demonstrated that MG53 can directly bind to FAK to regulate myogenesis in myoblast cells, indicating that FAK can serve as an essential target of MG53.^10^ However, no previous study has determined the roles of MG53 on FAK activation in endothelial cells and the consequent effects on endothelial cell physiological responses. In this study, we suggested that rhMG53 inhibits endothelial cell migration and tube formation through regulating FAK/Src/Akt/ERK1/2 signalling pathways.

## 2 MATERIALS AND METHODS

### 2.1 Chemicals and reagents

Primary human umbilical vein endothelial cells (HUVECs) and the endothelial cell medium were purchased from ScienCell Research Laboratories. Recombinant MG53 was obtained from Novoprotein. Growth factor–reduced Matrigel Matrix was purchased from BD Biosciences. Anti‐MG53 antibody used for Western blotting was from Abcam, and anti‐MG53 antibody used for immunostaining was from Atlas Antibodies. Antibodies to phosphorylated FAK^Y397^, FAK, phosphorylated Src^Y416^, Src, phosphorylated Akt^T308^, Akt, phosphorylated ERK1/2 and ERK1/2 were obtained from Cell Signaling Technology. Methyl‐β‐cyclodextrin (MβCD) was from Absin Bioscience. Endothelial marker isolectin B4 (IB4) was from Invitrogen. Pierce classic IP kit was from Thermo Scientific.

### 2.2 Cell culture

HUVECs were cultured in endothelial cell medium with 5% (v/v) foetal bovine serum (FBS), 1% (v/v) endothelia cell growth supplement and 1% (v/v) antibiotic solution. Cells used were passaged 4‐10 times.

### 2.3 Cell scratch wound healing assay

HUVECs, cultured in 24‐well plates, were allowed to grow to 90% confluence and scratched with a 200 μL pipette tip, followed by stimulation with rhMG53 (0, 5, 10 and 20 μg/mL) for 24 hours. Photomicrographs were taken immediately after the scratch and after rhMG53 treatment. ImageJ software was used to measure the change in scratch area over time.

### 2.4 Cell migration assay

HUVEC migration was also performed with transwell chambers with an 8.0 μm‐sized porous membrane. HUVECs (2 × 10^4^/well) were added to the upper chambers and treated with rhMG53 (0, 5, 10 and 20 μg/mL). As a chemoattractant, the endothelial cell medium containing 2% FBS was added to the lower chambers. After 24 hours, cells remaining in the upper chambers were removed and the cells that have migrated to the lower chambers were fixed with 4% paraformaldehyde, stained with 0.5% crystal violet, photographed with a microscope and counted.

### 2.5 Tube formation assay

HUVECs (1 × 10^5^/well) were seeded onto 24‐well plates, which were pre‐coated with growth factor–reduced Matrigel matrix (250 μL/per well), and exposed to 1% FBS growth medium containing rhMG53 (0, 5, 10 and 20 μg/mL) for 18 hours. Then, tube formation was photographed and the total tube length in 5 fields was quantified using ImageJ software.

### 2.6 Western blotting

HUVECs were lysed in ice‐cold RIPA lysis buffer (Beyotime) supplemented with 1% protease and phosphatase inhibitor cocktail (Thermo Scientific). Equal amounts of proteins were separated using SDS‐PAGE and transferred to polyvinylidene fluoride membrane. Among the different experiments, the membranes were blocked and incubated with primary antibodies for MG53 (1:1000), phosphorylated FAK^Y397^ (1:1000), phosphorylated Src^Y416^ (1:1000), phosphorylated Akt^T308^ (1:1000), phosphorylated ERK1/2 (1:1000), total FAK (1:1000), total Src (1:1000), total Akt (1:1000) and total ERK1/2 (1:1000) overnight at 4℃. Then, the membranes were exposed to horseradish peroxidase‐conjugated species‐specific respective goat IgG (Cell Signaling Technology) for 60 minutes at room temperature. The protein bands were visualized with chemiluminescence, and ImageJ software was used to measure the density of the bands.

### 2.7 Immunofluorescent staining

In order to investigate the time course of rhMG53 uptake, HUVECs were stimulated with rhMG53 (10 μg/mL) for 0, 15, 30 and 120 minutes. In order to determine the effects of MβCD on rhMG53 uptake, HUVECs were pre‐treated with MβCD (5 mM) for 1 hour, followed by stimulation with rhMG53 (10 μg/mL) for 3 hours. Then, the cells were fixed with 4% paraformaldehyde for 15 minutes at room temperature, permeabilized with 0.5% Triton X‐100 for 15 minutes at 4℃, blocked with 5% bovine serum albumin at 37℃ and incubated with a primary anti‐MG53 antibody (1:50) at 4℃ overnight. After washing with PBS, the cells were incubated with rhodamine‐conjugated secondary antibody (1:100) against the primary antibody applied and the nuclei were stained with 4’, 6‐diamidino‐2‐phenylindole (DAPI).

### 2.8 Immunoprecipitation

HUVECs were stimulated with rhMG53 (10 μg/mL) for 24 hours, and the protein samples were prepared. Classic IP kit was used to perform protein immunoprecipitation. In brief, equal amounts of protein were incubated with protein A/G plus agarose beads and anti‐FAK antibody or IgG overnight at 4℃, with gentle rotation. According to the IP kit's instruction, the immune complexes were captured and solubilized by 50 μL SDS sample buffer. Then, the immune complexes were subjected to SDS‐PAGE and immunoblotted with primary antibodies against MG53 and Src.

### 2.9 Animal models

Postnatal retinal angiogenesis model was used in this study. All animal care and experimental procedures were approved by the Animal Care and Use Committee of Southern Medical University. Postnatal C57BL/6J mice were injected intraperitoneally with vehicle control or rhMG53 at 6 mg/kg bodyweight from postnatal day 5 (P5) to P12.

### 2.10 Retina whole‐mount immunostaining

Mouse retina whole‐mount immunostaining was performed as previously described.^27^ In brief, the eyes were harvested and immediately fixed with 4% paraformaldehyde for 2 hours at room temperature. Then, the eyes were washed 3 times with ice‐cold PBS and the retinas were dissected and cut into four radial incisions under a stereomicroscope. After being fixed with 4% paraformaldehyde, permeabilized with 0.5% Triton X‐100 and blocked with 5% bovine serum albumin, the retinas were washed and incubated with endothelial marker IB4 (1:100) at 4℃ overnight. Following thorough washing, the retinas were transferred to the glass slide, flat‐mounted and sealed under a glass coverslip in mounting media.

### 2.11 Statistical analysis

One‐way ANOVA followed by post hoc comparison was performed to analyse the results, and all of the data are shown as mean ± standard deviation (SD). A *P* value of <.05 was considered as statistically significant.

## 3 RESULTS

### 3.1 rhMG53 directly enters endothelial cells

**FIGURE 1:**
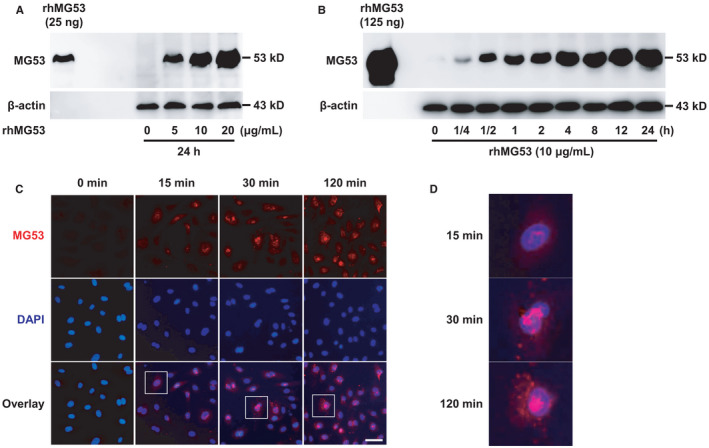
rhMG53 enters endothelial cells. A, HUVECs were exposed to rhMG53 (0, 5, 10 and 20 μg/mL) for 24 h. Then, the entry of rhMG53 into the endothelial cells was detected by Western blotting in total cell lysates. Representative images of 3 independent experiments are shown. B, HUVECs were exposed to rhMG53 (10 μg/mL) for 0, 0.25, 0.5, 1, 2, 4, 8, 12 and 24 h. Then, the rhMG53 entry into the endothelial cells was detected by Western blotting in total cell lysates. Representative images of 3 independent experiments are shown. C, HUVECs were stimulated with rhMG53 (10 μg/mL) for 0, 15, 30 and 120 min. Then, the uptake of rhMG53 by endothelial cells was identified by incubating with a primary anti‐MG53 antibody at 4℃ overnight, followed by immunostaining with a rhodamine‐conjugated secondary antibody (red). DAPI was used to label the nuclei (blue) and representative images of at least 3 independent experiments are shown. Scale bar is 50 μm. D, The enlarged images of the single cell in the white square are shown

MG53 is mainly expressed in striated muscle, and we fail to detect endogenous MG53 protein in HUVECs (Figure S1). To explore whether rhMG53 could act on endothelial cells, we firstly determined whether rhMG53, when added to the extracellular space, could directly enter the HUVECs. HUVECs were treated with rhMG53 (0, 5, 10 and 20 μg/mL) for 24 hours. Then, the MG53 protein in endothelial cells was detected with Western blotting. The results demonstrated that the MG53 protein bands clearly appeared after rhMG53 stimulation, indicating the direct entry of rhMG53 into endothelial cells (Figure 1A). To determine the time course of rhMG53 uptake, HUVECs were exposed to rhMG53 (10 μg/mL) for 0, 0.25, 0.5, 1, 2, 4, 8, 12 and 24 hours and the results showed that the entry of rhMG53 into endothelial cells occurred 15 minutes after it was added to the extracellular space (Figure 1B). Consistent with Western blotting results, the experiments involving immunofluorescence staining also confirmed that 15‐min incubation of HUVECs with rhMG53 resulted in protein uptake (Figure 1C,D). Taken together, these results indicate rhMG53 can directly enter into endothelial cells.

**FIGURE 2:**
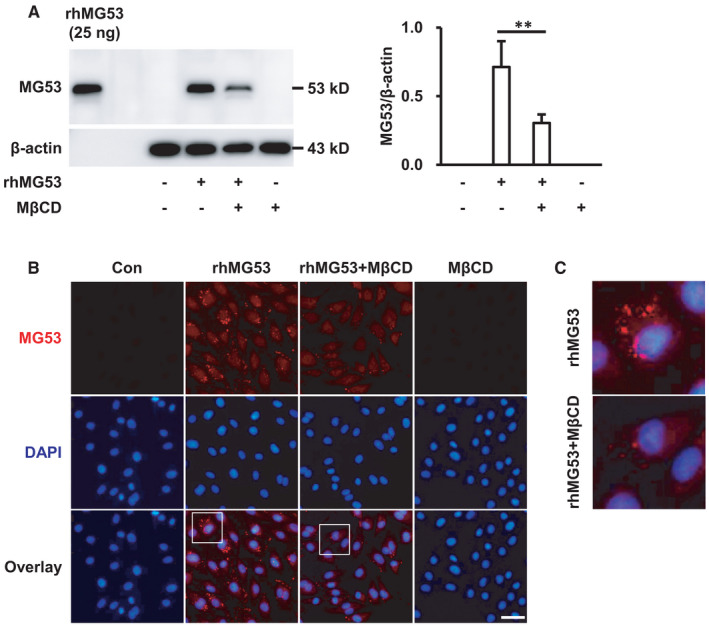
rhMG53 enters endothelial cells in a cholesterol‐dependent manner. HUVECs were pre‐treated with MβCD (5 mM) at 37℃ for 1 h, followed by exposure to rhMG53 (10 μg/mL) for 3 h. A, Then, the expression of MG53 was detected by Western blotting in total cell lysates. Representative images of 3 independent experiments are shown. **P < .01. B, The rhMG53 entry into cells was identified by incubating with a primary anti‐MG53 antibody at 4℃ overnight, followed by immunostaining with a rhodamine‐conjugated secondary antibody (red). DAPI was used to label the nuclei (blue) and representative images of at least 3 independent experiments are shown. Scale bar is 50 μm. C, The enlarged images of the single cell in the white square are shown

Furthermore, to investigate the specific mechanism of rhMG53 uptake, HUVECs were pre‐treated with MβCD (5 mM) for 1 hour to deplete membrane cholesterol,^28^, ^29^ followed by stimulation with rhMG53 (10 μg/mL) for 3 hours. The results showed that MβCD significantly decreased the uptake of rhMG53 by endothelial cells (Figure 2), suggesting that rhMG53 entered endothelial cells in a cholesterol‐dependent manner.

### 3.2 rhMG53 binds to FAK and inhibits FAK phosphorylation

**FIGURE 3:**
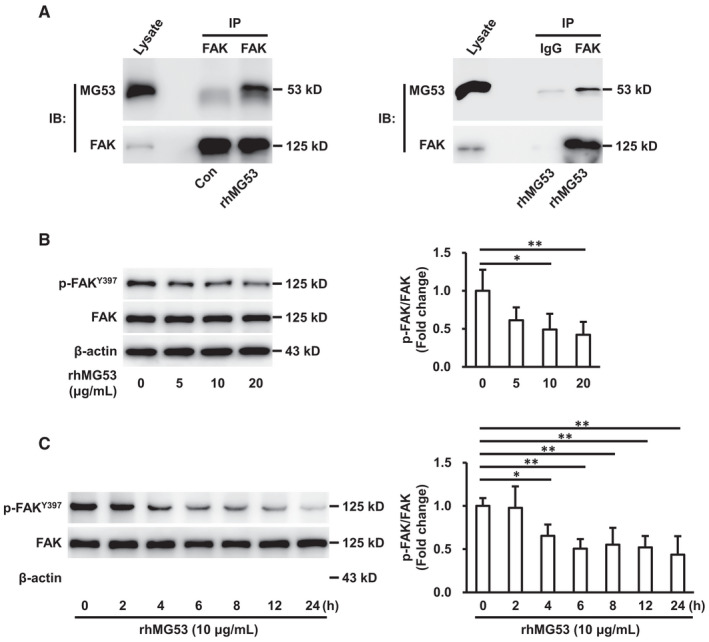
rhMG53 binds to FAK and inhibits FAK phosphorylation. A, HUVECs were stimulated with rhMG53 (10 μg/mL) or vehicle control for 24 h. Cell lysates were prepared and immunoprecipitated with anti‐FAK antibody or control IgG. Then, captured proteins were analysed by Western blotting with anti‐MG53 and anti‐FAK antibodies as indicated. Representative images of 3 independent experiments are shown. B, HUVECs were exposed to rhMG53 (0, 5, 10 and 20 μg/mL) for 24 h. Phosphorylation of FAK^Y397^ and total FAK were analysed by Western blotting, and representative images of three independent experiments are shown. The densitometric analysis of phosphorylated FAK^Y397^ normalized to total FAK was performed. All data shown are mean ± SD for 3 experiments and are expressed as fold changes. *P < .05; **P < .01. C, HUVECs were stimulated with rhMG53 (10 μg/mL) for 0, 2, 4, 6, 8, 12 and 24 h. Phosphorylation of FAK^Y397^ and total FAK were analysed by Western blotting and representative images of three independent experiments are shown. The densitometric analysis of phosphorylated FAK^Y397^ normalized to total FAK was performed. All data shown are mean ± SD for 3 experiments and are expressed as fold changes. *P < .05; **P < .01

Previous studies have reported that endogenous MG53 can interact with FAK in skeletal muscle cells.^10^ In order to investigate the role of rhMG53 uptake by endothelial cells, we suggested that rhMG53 may directly bind to FAK. To test this hypothesis, HUVECs were stimulated with rhMG53 (10 μg/mL) for 24 hours and the cell lysates were immunoprecipitated with anti‐FAK antibody or IgG and blotted with anti‐MG53 antibody. The results showed that MG53 significantly coimmunoprecipitated with FAK, but was not detected in IgG immunoprecipitates (Figure 3A), indicating that rhMG53 directly bound to FAK.

To explore the functional significance of rhMG53‐FAK interaction, HUVECs were stimulated with rhMG53 (0, 5, 10 and 20 μg/mL) for 24 hours and the phosphorylated and total FAK were detected by Western blotting. The results demonstrated that rhMG53 prominently decreased FAK phosphorylation at Y397 in a concentration‐dependent manner (Figure 3B). However, no significant difference in total FAK expression was seen (Figure S2). Furthermore, to determine the time course of rhMG53‐induced dephosphorylation of FAK, HUVECs were exposed to rhMG53 (10 μg/mL) for 0, 2, 4, 6, 8, 12 and 24 hours. The results displayed that rhMG53 remarkably decreased the phosphorylation of FAK at Y397 as early as in 4 hours and rhMG53 gradually induced FAK dephosphorylation in a time‐dependent manner (Figure 3C). All of the results suggest that rhMG53 directly binds to FAK and simultaneously inhibits FAK phosphorylation.

### 3.3 rhMG53 inhibits the activation of Src, Akt and ERK1/2

**FIGURE 4:**
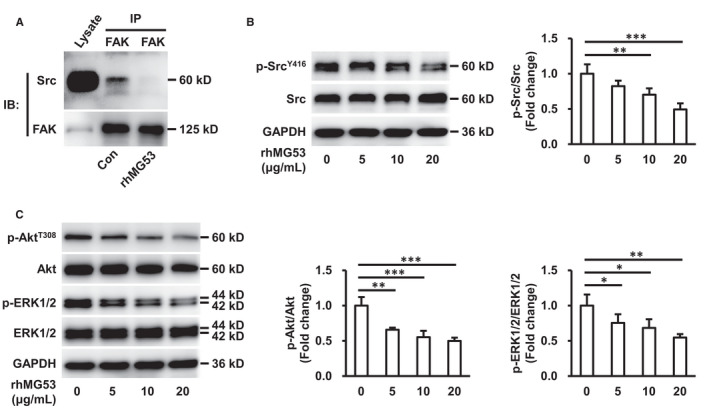
rhMG53 inhibits FAK‐Src interaction and Src, Akt and ERK1/2 activation. A, HUVECs were stimulated with rhMG53 (10 μg/mL) or vehicle control for 24 h. Cell lysates were prepared and immunoprecipitated with anti‐FAK antibody. Then, captured proteins were analysed by Western blotting with anti‐Src and anti‐FAK antibodies as indicated. Representative images of 3 independent experiments are shown. B and C, HUVECs were exposed to rhMG53 (0, 5, 10 and 20 μg/mL) for 24 h. Phosphorylation of Src^Y416^, Akt^T308^ and ERK1/2, and total Src, Akt and ERK1/2 were analysed by Western blotting and representative images of 3 independent experiments are shown. The densitometric analysis of phosphorylated Src^Y416^, Akt and ERE1/2 normalized to total Src, Akt and ERK1/2 was performed. All data shown are mean ± SD for 3 experiments and are expressed as fold changes. *P < .05; **P < .01; ***P < .001

Src is one of the important downstream signals of FAK. FAK autophosphorylation at Y397 induces the binding of Src to FAK, which not only phosphorylates additional sites on FAK leading to its full activation, but also activates Src.^30^ The mutually activation of FAK/Src complex further phosphorylates multiple proteins to regulate different cell functions.^24^, ^31^ In order to investigate the effects of rhMG53 on the downstream signalling pathways of FAK, the interaction between FAK and Src was firstly determined by immunoprecipitation. The results showed that rhMG53 (10 μg/mL) treatment for 24 hours significantly inhibited the FAK‐Src complex formation (Figure 4A). The activation of Src was also detected and the results demonstrated that rhMG53 remarkably reduced the Src phosphorylation at Y416 (Figure 4B). Moreover, the effects of rhMG53 on FAK/Src downstream signalling pathways, such as Akt and ERK1/2, were subsequently investigated and rhMG53 also significantly suppressed the phosphorylation of Akt and ERK1/2 (Figure 4C). As a whole, all of these results indicate that rhMG53 inhibits the activation of FAK/Src/Akt/ERK1/2 signalling pathways.

### 3.4 rhMG53 inhibits endothelial cell migration and tube formation

**FIGURE 5:**
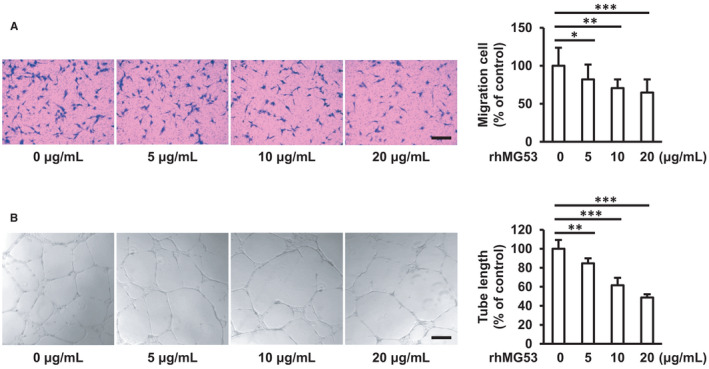
rhMG53 inhibits endothelial cell migration and tube formation. A, HUVECs were added to the upper chambers with an 8.0 μm‐sized porous membrane and stimulated with rhMG53 (0, 5, 10 and 20 μg/mL) for 24 h. Then, the cells that had migrated to the lower chambers were stained with crystal violet and counted. Representative images of cell migration are shown. Scale bar is 200 μm. Quantitative assessment of 3 independent experiments was performed. All data shown are mean ± SD and are expressed as % of control. *P < .05; **P < .01; ***P < .001. B, HUVECs were seeded onto 24‐well plates pre‐coated with Matrigel matrix (250 μL per well), followed by stimulation with rhMG53 (0, 5, 10 and 20 μg/mL) for 16 h. Representative images of tube formation are shown. Scale bar is 200 μm. The tube length was measured, and quantitative assessment of 3 independent experiments was performed. All data shown are mean ± SD and are expressed as % of control. **P < .01; ***P < .001

FAK/Src/Akt/ERK1/2 signalling pathways are involved in cell attachment, migration and proliferation, which play important roles in regulating angiogenesis.^24^, ^25^, ^32^ Therefore, we further explored the effects of rhMG53‐induced FAK/Src/Akt/ERK1/2 signalling inactivation on endothelial cell physiological responses. HUVECs were added to the upper chambers containing porous filters, followed by rhMG53 (0, 5, 10 and 20 μg/mL) stimulation for 24 hours. The results demonstrated that rhMG53 significantly decreased the number of cells that migrated to the lower chambers (Figure 5A). Consistent with the transwell cell migration results, rhMG53 also remarkably reduced the endothelial cell migration area in the scratch wound healing experiments (Figure S3). To investigate the effects of rhMG53 on tube formation, HUVECs were added to the 24‐well plates pre‐coated with growth factor–reduced Matrigel matrix and exposed to rhMG53 (0, 5, 10 and 20 μg/mL) for 18 hours. The results showed rhMG53 significantly inhibited endothelial cell tube formation (Figure 5B). Taken together, all of the findings indicate that rhMG53 inhibits angiogenesis in vitro. It is well known that vascular endothelial growth factor (VEGF) is the major angiogenic cell factor under physiological and pathophysiological conditions.^33^ The binding of VEGF to VEGF receptor‐2 (VEGFR‐2) leads to endothelial cell proliferation and migration and produces new blood vessels.^34^ In order to investigate whether the effects of rhMG53 on angiogenesis are associated with the changes of VEGF expression in HUVECs, HUVECs were stimulated with rhMG53 (10 μg/mL) for 24 hours, followed by an examination of VEGF and VEGFR‐2 expression with Western blotting. The results showed no significant difference in both VEGF and VEGFR‐2 expression (Figure S4), indicating that rhMG53 inhibited angiogenesis independent of VEGF/VEGFR‐2 signalling pathways.

### 3.5 rhMG53 inhibits angiogenesis in mouse retina

**FIGURE 6:**
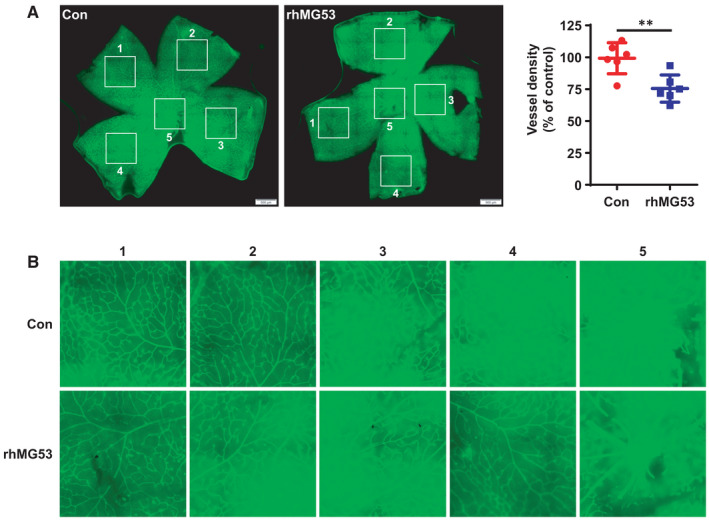
rhMG53 inhibits mouse retinal angiogenesis. A, Postnatal C57BL/6J mice were injected intraperitoneally with vehicle control or rhMG53 at 6 mg/kg bodyweight from P5 to P12. Mouse retinal vessels were observed by whole‐mount immunofluorescence staining for IB4 (green), and the vessel density was analysed. Representative images of retinal vessels are shown. **P < .01. B, The enlarged images of the retinal vessels in the white square are shown

To examine the significance of our findings in vivo, the effects of rhMG53 on retinal angiogenesis in postnatal mice were detected. Postnatal mice were injected intraperitoneally with rhMG53 (6 mg/kg) every day for 1 week. Then, the whole retina was cut into four radial incisions and the vessels were labelled by IB4 staining. The results demonstrated that the vessel density in rhMG53‐treated mouse retina significantly decreased (Figure 6). These data are consistent with our results in vitro and clarify the significance of anti‐angiogenesis effects of rhMG53 in vivo.

## 4 DISCUSSION

MG53 has been identified as a myokine/cardiokine secreted from cardiac and skeletal muscle in response to high glucose and insulin.^12^, ^20^ Increasing reports have focussed on the roles of MG53 in non‐striated muscle tissue,^15^, ^16^, ^17^, ^18^, ^19^, ^35^, ^36^ although it is expressed predominantly in cardiac and skeletal muscle. In the present study, the effects of rhMG53 on endothelial cells and angiogenesis were investigated. Our data demonstrated that rhMG53 entered endothelial cells in a cholesterol‐dependent manner, directly bound to FAK and resulted in the inactivation of FAK/Src/Akt/ERK1/2 signalling pathways, thereby inhibiting cell migration, tube formation and the consequent angiogenesis.

There have been no previous studies to investigate whether rhMG53 can act directly on the endothelial cells. In the present study, when rhMG53 was added to the extracellular space, Western blotting and immunofluorescence experiments revealed that rhMG53 directly entered endothelial cells. These results are consistent with previous reports which have shown that rhMG53 can be taken up into the cytosol by corneal fibroblasts^37^ and alveolar epithelial cells.^29^ Furthermore, published studies^28^, ^29^ and our experiments involving MβCD displayed the significant role of cholesterol in rhMG53 uptake, indicating a clathrin‐independent endocytosis in this process. Although further studies are required to confirm the specific pathway of rhMG53 uptake into the cells, on the basis of previous reports identifying an interaction between MG53 and caveolin, and the intimate interplay between MG53 and caveolar endocytosis,^38^, ^39^ we speculate that rhMG53 may enter cells via the caveolar endocytic pathway.

FAK has been demonstrated as a potential E3 ligase substrate for MG53. In C2C12 cell expressing endogenous MG53 and in HEK293 cells over‐expressing exogenous MG53, MG53 was determined to directly bind to FAK, which induced FAK ubiquitination and degradation.^10^ In agreement with these published data, our immunoprecipitation results also showed a significant interaction between rhMG53 and FAK. However, Western blotting analysis revealed that the total FAK expression did not change with rhMG53 treatment, which went against the notion that MG53 functions as E3 ligase to degrade FAK. Using a MG53 transgenic mouse line which achieved a sustained increase in circulating MG53, previous reports also found that sustained elevation of MG53 in the bloodstream had no significant effects on the protein level of FAK in mouse muscle.^13^ Our data and the published report both demonstrated the rhMG53 and circulating MG53 failed to degrade FAK via E3 ligase–mediated ubiquitination, which indicates that there may be some unknown differences between circulating MG53/rhMG53 and endogenous MG53. Meanwhile, it was surprising that the interaction between rhMG53 and FAK significantly decreased FAK phosphorylation in a time‐dependent and dose‐dependent manner. With immunoprecipitation assays in HEK293 cells, previous studies showed that MG53 interacted with the 4.1 protein/ezrin/radixin/moesin (FERM) and kinase domains of FAK.^10^ Both of these two domains play important roles in regulating FAK activation,^29^, ^40^, ^41^ so the binding of these domains with MG53 may be involved in the mechanism of rhMG53‐induced dephosphorylation of FAK.

The autophosphorylation of FAK at Y397 creates a binding site for Src via the SH2 domain, which induces the binding of Src to FAK and leads to activation of Src.^42^, ^43^ Growing evidence has demonstrated that the dephosphorylation of FAK at Y397 disrupted Src activation. Consistent with these data, our results also showed that accompanied by the dephosphorylation of FAK, rhMG53 significantly inhibited the interaction between FAK and Src and decreased the phosphorylation of Src at Y416. Furthermore, our results also showed rhMG53 dramatically retrained the activation of downstream signalling pathways of FAK/Src, such as Akt and ERK1/2. Although few previous studies have focussed on the effects of rhMG53 on FAK‐Src interaction and Src activation, in agreement with our results, the published data have shown that knockdown of MG53 remarkably promoted the phosphorylation of Akt in SCC25 cells,^44^ indicating the activation of Akt may be inhibited by MG53. Moreover, previous studies have also demonstrated that both endogenous MG53 and rhMG53 significantly inhibited insulin‐induced Akt signalling pathway activation,^8^, ^9^, ^12^ which is at least partly consistent with the findings in the present study. However, in cardiac ischaemia/reperfusion model, endogenous MG53 has been shown to participate in cardioprotection via activating Akt and ERK1/2,^6^, ^7^ which are opposite to our results. But, when rhMG53 was applied, it resulted in Akt phosphorylation only at the infarct zone of hearts induced by ischaemia/reperfusion and had no effects on ERK1/2 activation.^45^ Taken our results and the published data into consideration, the effects of MG53 on Akt and ERK1/2 signalling pathways may depend on cell types and animal models and further studies are needed to better clarify the underlying molecular mechanisms.

The mutual activation of FAK/Src and its downstream signalling pathways contributes to integrin‐dependent and growth factor–dependent migration and angiogenesis. Consistent with the inhibitory effects of rhMG53 on FAK/Src/Akt/ERK1/2 signalling, our experiments involving endothelial cell physiological response measurements demonstrated that rhMG53 significantly decreased cell migration and tube formation in vitro. In agreement with the cell culture results, the retinal angiogenesis assay in postnatal mice also showed significant anti‐angiogenesis effects of rhMG53 in vivo. Previous reports have shown that at day 7 post–alkaline injury, *mg53^−/−^* corneas had more vascularization and rhMG53 treatment remarkably decreased alkaline injury‐induced cornea angiogenesis in *db/db* mice,^14^, ^37^ both of which support the findings in the present study. However, a limitation of our in vivo experiments was that we did not definitively prove the inhibitory effects of rhMG53 on FAK, Src, Akt and ERK1/2 phosphorylation. Nevertheless, our findings in vivo and the published data complement and support the significance of our in vitro experiments, which demonstrated that rhMG53 inhibited angiogenesis via regulating the activation of FAK/Src/Akt/ERK1/2 signalling pathways. Further studies will be needed to clarify the significance of our newly reported anti‐angiogenesis effects of rhMG53 in other disease models.

In summary, the findings in the present study advance our understanding for rhMG53 in the regulation of endothelial cell function. We show that rhMG53 inhibits angiogenesis with mechanisms involving entry into HUVECs in a cholesterol‐dependent manner, directly binding to FAK, uncoupling FAK‐Src interaction and consequently inactivation of Src/Akt/ERK1/2 signalling pathways. These data indicate that the elevation of serum MG53 may contribute to the underlying molecular mechanisms of diabetic microangiopathy. In addition, our results shall provide a base for the potential application of rhMG53 to treat diseases with excessive angiogenesis.

## CONFLICTS OF INTEREST

The authors declare no conflict of interest.

## AUTHOR CONTRIBUTIONS

**Jinling Dong:** Formal analysis (equal); Investigation (lead); Writing‐original draft (equal); Writing‐review & editing (equal). **Haiyan Zhou:** Formal analysis (equal); Investigation (equal). **Yongjie Li:** Investigation (supporting); Methodology (equal); Visualization (equal). **Rong Li:** Investigation (supporting); Methodology (supporting); Project administration (supporting). **Ni Chen:** Investigation (supporting); Methodology (supporting). **Youkun Zheng:** Project administration (supporting); Software (equal); Visualization (equal). **Xin Deng:** Methodology (supporting); Software (equal). **Mao Luo:** Formal analysis (equal); Project administration (supporting). **Jianbo Wu:** Project administration (supporting); Supervision (equal); Writing‐review & editing (equal). **Liqun Wang:** Data curation (lead); Formal analysis (lead); Funding acquisition (lead); Project administration (lead); Supervision (equal); Writing‐review & editing (equal).

## Supporting information

Supplementary Material

## Data Availability

The data that support the findings of this study are available from the corresponding author upon reasonable request.
