# Supplementary material for: MG53 inhibits angiogenesis through regulating focal adhesion kinase signalling
Source: J Cell Mol Med. 2021 Jul 9;25(15):7462–71. doi: 10.1111/jcmm.16777 (PMC8335693; doi:10.1111/jcmm.16777)
Supplement: Supplementary file 1 — Supplementary Material [file JCMM-25-7462-s001.docx]

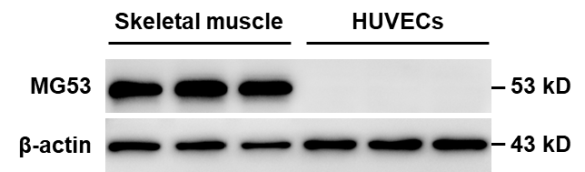


**Figure S1** MG53 is not expressed in endothelial cells. Proteins from mouse skeletal muscle and HUVECs were prepared and the expression of MG53 was detected with western blotting.


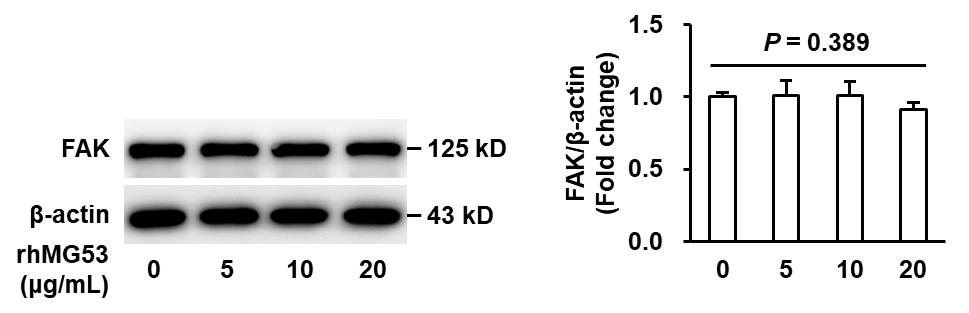


**Figure S2** rhMG53 has on effect on total FAK expression in HUVECs. HUVECs were stimulated with rhMG53 (0, 5, 10 and 20 μg/mL) for 24 h. Cell lysates were prepared and the expression of total FAK were measured with western blotting. Representative images of 3 independent experiments are shown and densitometric analysis of FAK normalized to β-actin was performed.


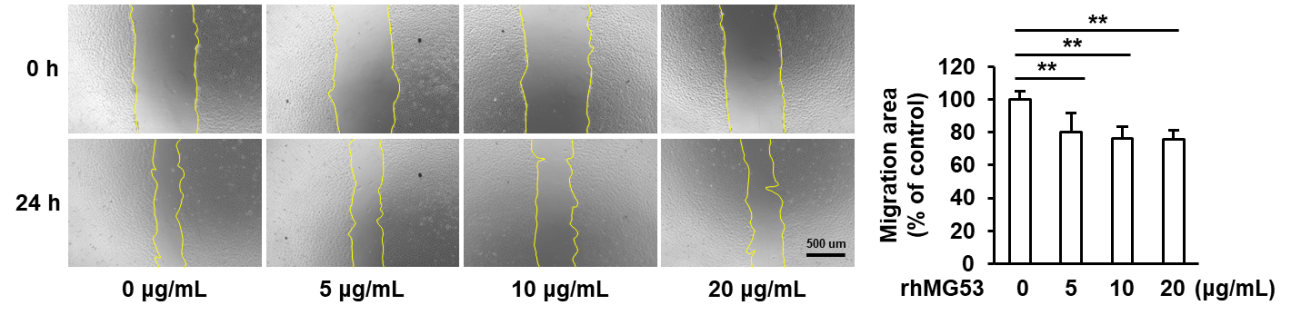


**Figure S3** rhMG53 decreases endothelial cell migration area. HUVECs were seeded onto 24-well plates and grown to 90% confluence. Then the cells were scratched with a 200 μL pipette tip, followed by stimulating with rhMG53 (0, 5, 10 and 20 μg/mL) for 24 h. Photomicrographs were taken immediately after the scratch and after rhMG53 treatment. Representative images of 3 independent experiments are shown. The cell migration area was calculated and quantitative assessment of 3 independent experiments was performed. ***P* < .01


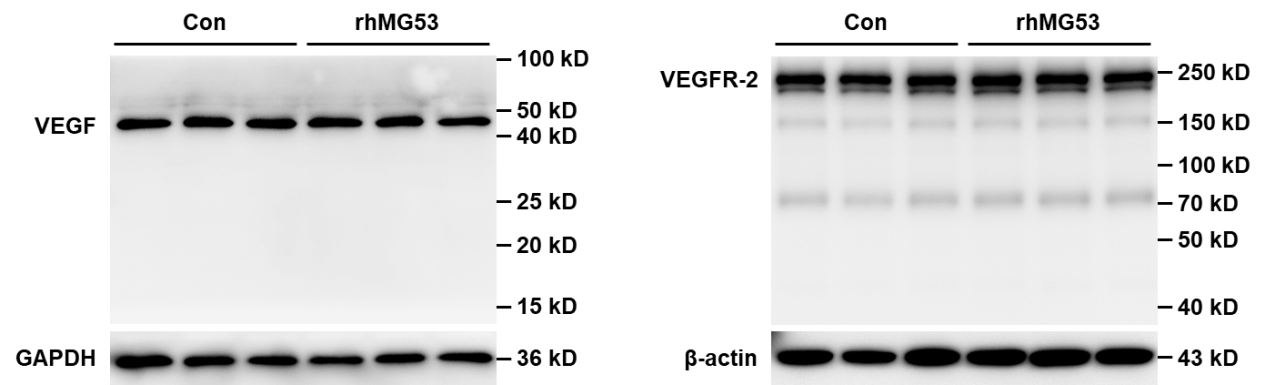


**Figure S4** rhMG53 has no effect on VEGF and VEGFR-2 expression in endothelial cells. HUVECs were stimulated with rhMG53 (10 μg/mL) for 24 h. Cell lysates were prepared and the expression of VEGF and VEGFR-2 were measured with western blotting.
